# Activation of the interleukin-23/Th17 axis in major depression: a systematic review and meta-analysis

**DOI:** 10.1007/s00406-024-01864-2

**Published:** 2024-07-16

**Authors:** Calum D. Moulton, Mantas Malys, Christopher W. P. Hopkins, Anna S. Rokakis, Allan H. Young, Nick Powell

**Affiliations:** 1 Department of Psychological Medicine, Institute of Psychiatry, Psychology & Neuroscience, King’s College London, London, SE5 8AF UK; 2 Department of Psychiatry, Division of Brain Sciences, Imperial College London, London, UK; 3 St Mark’s Hospital, London, UK; 4 Berkshire Healthcare NHS Foundation Trust, Thatcham, Berkshire UK; 5 Faculty of Medicine, Department of Metabolism, Digestion and Reproduction, Imperial College London, London, UK

**Keywords:** Depression, Interleukin-23, Th17 cells, Inflammation, Meta-analysis

## Abstract

The interleukin-23/Th17 axis is a promising modifiable target for depression. However, its association with depression has not been systematically evaluated. We systematically searched four databases (EMBASE, Web of Science, Pubmed and PsycINFO) for studies comparing patients with major depression and healthy controls for plasma/serum levels of Th17 cells and their canonical cytokines (interleukin-17A [IL-17A], IL-22, granulocyte macrophage colony stimulating factor [GM-CSF]). We also compared counts of Th1, Th2 and Th9 cells between depressed/non-depressed patients and their respective canonical cytokines. We performed random-effects meta-analysis of the standardised mean difference (SMD) in immune measures between groups. Risk of bias was assessed using the Newcastle–Ottawa scale. Of 3154 studies screened, 36 studies were included in meta-analysis. Patients with depression had elevated IL-17A compared to controls (SMD = 0.80 [95% CI 0.03 to 1.58], p = 0.042), an association moderated by antidepressant use (Z = 2.12, p = 0.034). Patients with depression had elevated GM-CSF (SMD = 0.54 [95% CI 0.16 to 0.91], p = 0.0047), and a trend towards higher Th17 counts (SMD = 0.44 [− 0.01 to 0.88], p = 0.052). Whilst the Th2-associated cytokine IL-5 was elevated in depression (SMD = 0.36 [95% CI 0.05 to 0.66], p = 0.02), Th2 cell counts (p = 0.97), Th1 cell counts (p = 0.17) and interferon-γ (p = 0.22) were not. Data for Th9 cells, IL-9 and IL-22 were insufficient for meta-analysis. Respectively, 22, 25 and 5 studies were good, fair and poor in quality. Patients with major depression show peripheral over-activation of the IL-23/Th17 axis. Future interventional studies should test whether this is a modifiable target for depression.

**Supplementary Information:**

The online version contains supplementary material available at 10.1007/s00406-024-01864-2.

## Introduction

Major depressive disorder (MDD) is the leading cause of disability internationally [1]. Despite advances in the treatment of depression, around 30% of patients do not respond to two consecutive antidepressant treatments [2], emphasising the need to identify novel treatment targets for depression.

For over 25 years, the immune system has been proposed as a potential causal pathway to depression [3]. This has been supported by a range of observational findings [3, 4], and inflammation is particularly associated with depression that does not respond to standard antidepressants [5]. To date, however, clinical translation of these findings has been limited. Broad-based anti-inflammatory approaches, such as antibiotics and non-steroidal anti-inflammatory drugs, have produced mixed findings [6–9]. More targeted approaches, such as anti-IL-6 and anti-TNF agents, have shown limited promise [7, 10, 11] and are associated with potentially serious side-effects [12]. There is therefore a need to identify novel modifiable immune pathways associated with depression.

The interleukin-23-Th17 axis is a promising candidate pathway. Interleukin-23 (IL-23) activates effector lymphocyte populations, including pathogenic T-helper-17 (Th17) cells, triggering release of pro-inflammatory cytokines including IL-17A and IL-22 [13]. The IL-23/Th17 axis already provides targets for multiple licensed treatments for immune-mediated inflammatory diseases (IMIDs), including inflammatory bowel disease (IBD) and psoriasis. These treatments include ustekinumab (an antagonist of the p40 subunit of IL-23 shared with IL-12), and more recently, monoclonal antibodies that target the specific p19 subunit of IL-23, including risankizumab, mirikizumab, guselkumab and tildrakizumab. Unlike other biologics targeting inflammatory cytokines, such as anti-TNF or anti-IL6, agents targeting IL23 are considered lower risk, with side effect profiles not significantly different from placebo [14–17]. Notably, in a previous meta-analysis in psoriasis, ustekinumab was the only biologic therapy to improve depressive symptoms after accounting for changes in physical disease [18]. Subsequent psoriasis trials have found that IL-23-specific antagonists (those targeting the IL-23p19 subunit) improve depression even more than ustekinumab [19].

Outside of IMIDs, a growing number of studies have reported an association between IL-23/Th17-associated cytokines and depression. Patients with major depression have been found to have higher Th17 cell counts than controls [20], as well as elevated peripheral cytokines on the Th17 axis, such as IL-17A [21] and granulocyte macrophage colony stimulating factor (GM-CSF) [22]. Meanwhile, there is increasing evidence that the IL-23/Th17 axis may cause neuroinflammation and depression. IL-22 and its receptor may be expressed in human brain [23] and IL-23 is required for T-cells to active resident microglia and recruit T-cells and macrophages to the central nervous system [24].

Collectively, these findings suggest that the IL-23/Th17 axis is a promising candidate pathway to target in depression, which warrants further investigation. To date, however, the role of the IL-23/Th17 axis in depression has not been systematically evaluated. We therefore performed a systematic review and meta-analysis to test the association between major depression and activation of the IL-23/Th17 axis, as measured using plasma/serum markers on this axis. To assess specificity for the IL-23/Th17 axis in depression over other immune pathways, we also captured key measures on the Th1, Th2 and Th9 axes in relation to depression. We hypothesised that depression would be associated with activation of the Th17 axis but not the Th1, Th2 and Th9 axes.

## Methods

### Design

The development of this systematic review was directed by the standards of the Preferred Reporting Items for Systematic Review and Meta-Analysis (PRISMA) Statement [25]. Studies were analysed to ensure that they met the review criteria and those with adequate data (at least 3 comparable studies of the same immune outcome) were pooled for meta-analysis.

### Literature search

We systematically searched EMBASE, Web of Science, Pubmed and PsycINFO from inception to 31st December 2023 for studies comparing key measures on the Th17, Th1, Th2 and Th9 axes (see below) between patients with major depression and healthy controls. Our literature search was as follows: (interleukin-17 OR il17 OR il-17 OR il-17A OR interleukin-22 OR il-22 OR il22 OR interleukin-23 OR il-23 OR il23 OR granulocyte macrophage colony stimulating factor OR gm-csf OR gmcsf OR th17 OR t-helper-17 OR cxcl-1 OR cxcl1 OR th1 OR th2 OR th9 OR interferon-gamma OR IFN-gamma OR IFN-γ OR interferon-γ OR interleukin-5 OR il5 OR IL-5 OR inerleukin-13 OR il13 OR il-13 OR interleukin-9 OR IL-9 OR il9) AND (depression OR depressed OR depressive). We excluded animal search terms such as mouse and mice.

### Study selection

We included studies that: (1) were cross-sectional or longitudinal cohort in design; (2) recruited adult human participants meeting clinical criteria (DSM or ICD) for a depressive disorder; (3) recruited a healthy control group; (4) sampled peripheral blood/serum; and (5) measured a candidate marker of the IL-23 axis (see below) or interferon (IFN)-γ, interleukin-5 (IL-5), IL-13 or IL-9 using an ELISA or validated cytokine array, such as a Luminex. Studies were excluded if they: (1) were interventional in design (due to the highly selective samples in such studies); (2) used a paediatric sample (< 18 years of age); (3) recruited patients with a comorbid medical or physiological condition potentially affecting inflammatory levels, such as inflammatory arthritis, malignancy or pregnancy; (4) did not provide data in which mean and/or standard deviation could be calculated; (5) presented data already transformed with non-logarithmic transformations (e.g. square-root transformation); (6) presented only genetic results, e.g. mendelian randomisation studies; (7) presented non-original or non-peer-reviewed data, e.g., reviews or conference abstracts; or (8) presented data only on stimulated cytokine measures (e.g. lipopolysaccharide-stimulated cells). Titles, abstracts and full texts meeting inclusion criteria were sequentially examined, before relevant studies were analysed for data extraction independently by two authors (MM and ASR); any disagreements were resolved through discussion with a third author (CDM). Reference lists of included publications were also hand-searched. Where a sample demonstrated overlap between different papers, we excluded the smaller sample.

### Data extraction

Co-primary outcomes of interest were the following plasma/serum measures on the IL-23/Th17 axis: IL-23; its key effector T-cell population (Th17 absolute cell counts); its key effector cytokines (IL-22, IL-17A and GM-CSF); and key effector chemokine (CXCL1). To assess specificity of the Th17 axis for depression, we also studied Th1 and Th2 cell counts between patients with depression and healthy controls, as well as the canonical cytokines for the Th1 axis (IFN-γ), Th2 axis (IL-5 and IL-13) and Th9 axis (IL-9) [26]. In addition to the mean and standard deviation (SD) of each outcome, we extracted data on study design, origin, setting, number of patients, percentage of women, definition of depression, type of immune assay, and a priori matching between cases and controls.

### Quality assessment

Study quality was independently assessed by two authors (CWPH, MM) using a modification of the Newcastle–Ottawa Scale [27], which compares (1) adequacy of case definition (max 1 point); (2) representativeness of cases (1 point); (3) selection of controls (1 point); (4) definition of controls (1 point); (5) Comparability of cases and controls (2 points); (6) measurement of exposure, i.e. immune assay and protocol (2 points). As before, these scores were converted to good, fair and poor ratings according to Agency for Healthcare Research and Quality standards as follows: Good – 3 or 4 stars in Selection AND 1 or 2 in Comparability AND 1 or 2 in Exposure; Fair – 2 stars in Selection AND 1 or 2 in Comparability AND 1 or 2 in Exposure; Poor – 0 or 1 in stars in selection OR 0 in Comparability OR 0 or 1 in Exposure. The full breakdown of quality assessment is included as supplementary material.

### Statistical analysis

Where the same immune measure was reported by 3 or more studies, we used STATA 18 to calculate the standardised mean difference (SMD) between depressed and control groups, and pooled effect estimates were calculated using a random-effects model. In the meta-analysis, we included only studies where there were no differences in age or gender distribution between depressed and non-depressed groups. We further excluded from the meta-analysis any studies where there were known body mass index (BMI) differences between groups, unless the findings were adjusted for BMI. Heterogeneity between studies was quantified by calculating the *I*^*2*^ statistic, in which values of 30–60% may represent moderate heterogeneity; 50–90% substantial heterogeneity; and 75–100% considerable heterogeneity [28]. We explored moderate and high heterogeneity in subgroup analysis stratified by current prescription of antidepressant medication. Where there were 10 or more studies, we used random-effects meta-regression to test current antidepressant treatment (in any patients) as a moderator of the association between depression and immune measures. We also performed the following sensitivity analyses: (i) excluding small studies (n < 40 patients total) and (ii) excluding poor-quality studies. Where more than 10 studies were included in a meta-analysis, publication bias was assessed using Funnel plots and Egger’s test.

## Results

**Fig. 1:**
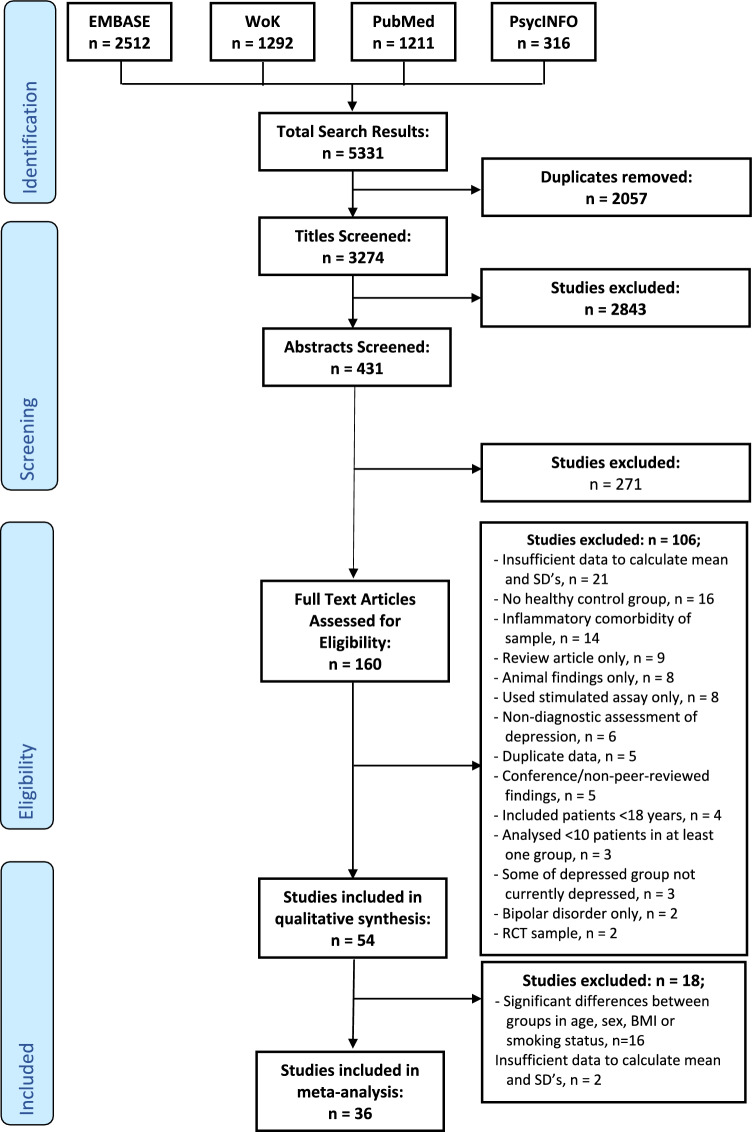
PRISMA flow diagram of literature search

Of 3154 studies screened, 54 studies were included in the qualitative synthesis, of which 36 studies were included in the meta-analysis. Figure 1 shows the PRISMA flow diagram of the search. Characteristics of included studies are summarised in Table 1.

**Table 1:** Characteristics of studies included in systematic review

First author	Year	No. depressed	No. cntrls	Country	Matching	Age of cases vs controls (years)			Sex (% female)			BMI			Immune assay	Definition of depression
						Dep	Cont	P-value	Dep	Cont	P-value	Dep	Cont	P-value		
Al Hakeim	2020	140	40	Iraq	None	39	40.3	0.47	44	45	0.94	27.6	27.7	0.95	ELISA	MDD—semi-structured interview based on ICD-10 criteria
Almulla	2023	71	40	Iraq	None	31.2	32.1	0.60	42.3	42.3	0.98	24.3	26.0	0.029	Bio-Plex Multiplex Immunoassay	MDD—clinician assessment using DSM-5 criteria
Alves	2020	139	100	Brazil	None	43	31	0.001	58.3	44	0.03	M	M	M	Th1 Human Cytokine Kit	MDD—MINI using DSM-IV criteria
Baek & Park	2013	80	80	South Korea	Age, sex	44.9	44.5	0.641	73.8	65	0.14	22.6	22.7	0.98	ELISA	MDD—clinician assessment using DSM-IV criteria
Bayes	2024	80	81	Australia	Age	36.2	33.4	0.12	62.5	50.6	0.17	26.8	24.4	0.008	Luminex	MDD—clinician assessment using DSM-5 criteria
Becking	2018	90	165	Nether-lands, Belgium, Germany	None	40.8	40.3	NS	55.6	58.5	NS	26.6	23.5	< 0.05	Unlisted	MDD—SCID using DSM-IV criteria
Blzniewska-Kowalska	2020	95	30	Poland	None	47.2	37.7	0	71.6	76.7	0.59	25.1	24.5	0.69	ELISA	Depressive episode or recurrent depressive disorder using ICD-10 criteria
Cassano	2017	118	118	USA	Age, sex	42.1	42.1	0.98	55	55	1	25	26.3	0.02	Luminex	MDD—SCID using DSM-IV criteria
Chen	2018	91	90	Taiwan	Age, sex	41.4	38.9	0.17	61.8	54.4	0.33	22.3	22.3	1	Multiplex	MDD—clinician assessment using DSM-5 criteria
Chen	2011	40	30	China	Age, sex, ethnicity	34.7	33.1	0.59	37.5	40	0.83	M	M	M	ELISA	MDD—clinician assessment using DSM-IV criteria
Choi	2021	41	59	South Korea	None	41	38.5	0.43	73.2	62.7	0.24	22.9	22.8	0.68	Luminex	MDD—MINI using DSM-IV criteria
Daria	2020	120	100	Bangladesh	Age, sex, BMI	32.2	33.2	0.426	56	55	0.57	25.3	24.6	0.24	ELISA	MDD—clinician assessment using DSM-5 criteria
Davami	2016	41	40	Iran	Age	35.6	36.3	0.75	73.2	60	0.21	24.3	22.9	0.41	ELISA	MDD—clinician assessment using DSM-IV criteria
Eidan	2019	60	30	Iraq	None	33	31.1	0.21	33.3	56.7	0.043	24.9	24.1	0.32	ELISA	MDD—clinician assessment using DSM-IV criteria
Elomaa	2012	58	58	Finland	None	53.4	53.2	0.91	69	69	1	27.2	27.1	0.97	Luminex	MDD—clinician assessment using DSM-IV criteria
Fanelli	2019	76	76	South Korea	None	68.1	66.2	< 0.05	78.9	56.6	< 0.05	25.1	25	0.85	Multiplex	MDD—MINI using DSM-IV criteria
Fornaro	2013	30	32	Italy	None	48.3	45.2	NS	80	75	NS	M	M	M	ELISA	MDD—clinician assessment using DSM-IV criteria
Ghosh	2020	53	53	India	None	29.2	30.9	0.26	40	36	0.69	M	M	M	Unlisted	MDD—ICD-10 assessed using HAM-D
Grosse	2016	71	71	Germany	Age, sex	33	31	0.51	62	66	0.6	25	23	0.045	ELISA	MDD—MINI using DSM-IV criteria
Grosse	2016a	40	40	Nether-lands	Age, sex	52	49	0.15	60	50	0.37	M	M	M	FACS (cell only)	MDD—SCID using DSM-IV criteria
He	2020	44	22	China	None	25.2	25.6	0.45	59	54.5	0.73	M	M	M	ELISA	MDD—clinician assessment using DSM-IV criteria
Hernandez	2008	31	22	Mexico	None	32	30.8	0.59	71	68.2	0.83	24.2	23.6	M	ELISA	MDD—MINI (Spanish) using DSM-IV criteria
Ho	2017	12	20	Taiwan	Age, sex, BMI	23.2	25.2	NS	0	0	1	23.4	23.4	1	Luminex	MDD—clinician assessment using DSM-IV criteria
Hocaoglu	2012	30	30	Turkey	Age, sex, education	38	30	0.008	80	46.7	0.007	M	M	M	ELISA	MDD—SCID using DSM-IV criteria
Hosseini	2017	37	15	Iran	None	39.1	38.2	0.81	73	73	1	M	M	M	ELISA	DSM-IV criteria for MDD using clinician assessment
Hughes	2012	39	39	Ireland	Age, sex	41.9	37.1	0.077	59	56.4	0.82	25.3	23.9	0.098	ELISA	MDD—clinician assessment using DSM-IV criteria
Kageyama	2018	109	29	Japan	None	46	40.8	0.11	46.8	51.7	0.91	M	M	M	Multiplex and ELISA	MDD—clinician assessment using DSM-IV criteria
Kakeda	2018	40	47	Japan	None	46.6	40.7	0.06	50	27.7	0.05	M	M	M	Multiplex	MDD—clinician assessment using DSM-IV criteria
Kim	2021	50	50	Korea	Age	24.2	24.8	0.39	54	52	0.84	22.7	21.8	M	ELISA	MDD—SCID using DSM-IV criteria
Kim	2013	26	28	South Korea	Age, sex	36.7	35.6	0.864	80.8	82.1	0.9	24.3	22.86	0.36	ELISA	MDD—SCID using DSM-IV criteria
Kim A	2007	48	63	South Korea	Age, sex	38	42.3	0.063	62.5	74.6	0.17	21.9	21.2	M	ELISA	MDD—clinician assessment using DSM-IV criteria
Kiraly	2017	33	26	USA	Age, sex, BMI	44.8	39	0.082	42.4	53.8	0.3	28.2	27.2	0.19	Multiplex	MDD/TRD—SCID and non-response ≥ 2 antidepressant trials
Lin	2018	100	100	China	None	36.5	35.3	0.17	58	62	0.66	21.8	22.6	0.19	MTT & ELISA	MDD—clinician assessment using DSM-IV criteria
Mao	2018	62	64	China	Age, sex, BMI, edu-cation	30.8	31.8	0.29	51.6	53.2	0.72	22.6	23.1	0.4	ELISA	MDD—SCID using DSM-IV criteria
Mao	2022	40	40	China	Age, sex	34.9	36.6	0.52	60.0	67.5	0.49	M	M	M	ELISA	MDD—clinician assessment using DSM-5 criteria
Marques-Deak	2007	46	36	Brazil	Age	39.4	37	0.28	100	100	1	26	24.7	0.23	ELISA	MDD—SCID using DSM-IV criteria
Pavon	2006	33	33	Mexico	Age, sex, ethnicity, socio-economic class	33.6	32.3	0.65	84.8	84.8	1	M	M	M	FACS, ELISA	MDD—SCID using DSM-IV criteria
Pedraz-Petrozzi	2020	43	51	Germany	Age, sex	25		0.19	72.1	62.7	0.1	23.7	22.7	0.57	ELISA	MDD—clinician assessment using DSM-IV criteria
Saraykar	2018	74	55	Brazil	None			0.28			0.53			0.56	Luminex	MDD—MINI using DSM-IV criteria
Schiweck	2020	202/153	212/153	Germany	Age, sex	37.8	37.8	0.96	63	63	1	25.1	23.8	0.007	FACS	MDD—MINI using DSM-IV criteria
Schmidt	2018	37	175	Germany	None	39.6	36.6	0.2	48.6	64.6	0.1	32	35.3	0.13	Bioplex	MDD—SCID using DSM-IV criteria
Shelton	2015	135	50	USA	None	45	38.4	0.001	65.9	80	0.06	30.9	30.4	0.71	Multiplex	MDD—MINI using DSM-IV criteria
Simon	2008	49	49	USA	Age, sex	41.7	41.7	NS	40.8	42.9	NS	M	M	M	Human 22-Plex	MDD—SCID using DSM-IV criteria
Spanem-berg	2014	33	54	Brazil	None	50.3	47.4	0.2	84.8	74.1	0.33	26.8	M	M	Flow cytometry Human Cytokine kit	MDD—MINI (Spanish) using DSM-IV criteria
Straw-bridge	2019	36	36	UK	Age, sex, BMI	53.8	54.5	NS	58	58	1	29.1	28.2	NS	Multiplex	MDD—MINI using ICD-10 criteria and TRD according to MSM
Suzuki	2017	54	56	USA	Age, sex	34.3	32.1	NS	75.9	75	NS	29.6	26.4	< 0.05	BD LSRII 4-laser flow cytometer	MDD—clinician assessment using DSM-IV criteria
Syed	2018	171	64	USA	None	39.4	45	0.001	66.1	46.9	0.007	28.8	29.2	0.73	Multiplex	MDD—assessment not listed
Wong	2018	68	18	USA	None	34.2	34.6	0.55	75	66.7	0.5	28.7	29	M	Multiplex	MDD—clinician assessment using DSM-IV criteria
Young	2016	35	25	USA	None	37.3	35.1	0.73	62.9	60	0.72	27.1	26.5	0.67	Mesoscale Discovery platform + ELISA	MDD—SCID using DSM-IV criteria
Zincir	2016	50	30	Turkey	None	33	31.2	0.23	64	66.7	0.8	M	M	M	ELISA	MDD/TRD—Diagnoses of TRD via SCID DSM-IV-TR
Zhou	2021	66	30	China	Age, sex	M	M	NS	M	M	NS	22.7	M	M	Luminex	MDD—clinician assessment using DSM-5 criteria, HAM-D > 17, failure of two adequate antidepressant trials
Zoga	2014	40	40	Greece	Age, BMI, meno-pausal status	51.1	52.3	0.58	100	100	1	26.6	26.4	0.85	ELISA	MDD—clinician assessment using DSM-IV criteria

*BMI* body mass index, *Cont *controls, *DSM* diagnostics and statistical manual, *ELISA* enzyme linked immunosorbent assay, *FACS* fluorescence-activated cell sorting, *HAM-D* Hamilton depression rating Scale, *M* missing, *MDD* major depressive disorder, *MINI* Mini neuropsychiatric interview, *NS* not significant, *SCID* structured clinical interview for DSM, *TRD* treatment-resistant depression

### Quality assessment

22 studies were of good quality, 25 were of fair quality and 5 were of poor quality (Supplementary Table 1).

## Th17 measures

### IL-17A

**Fig. 2:**
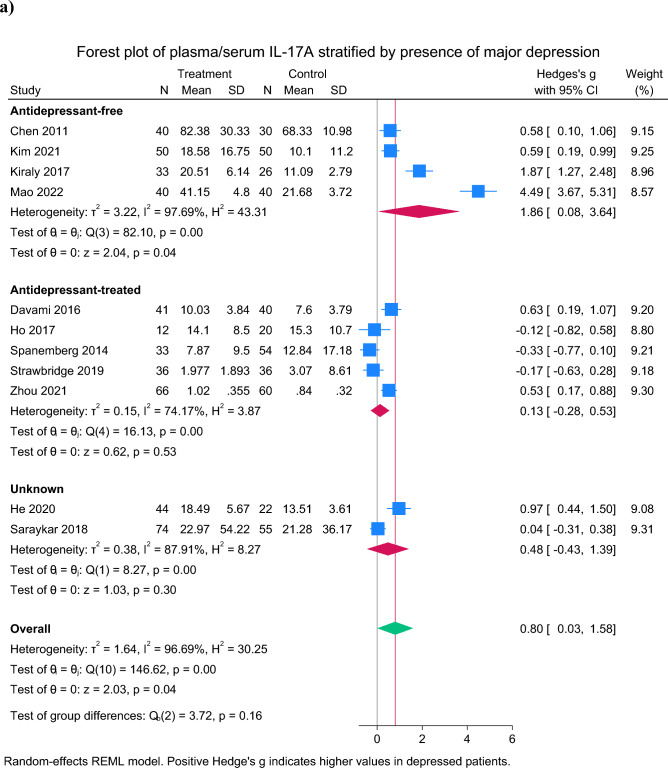

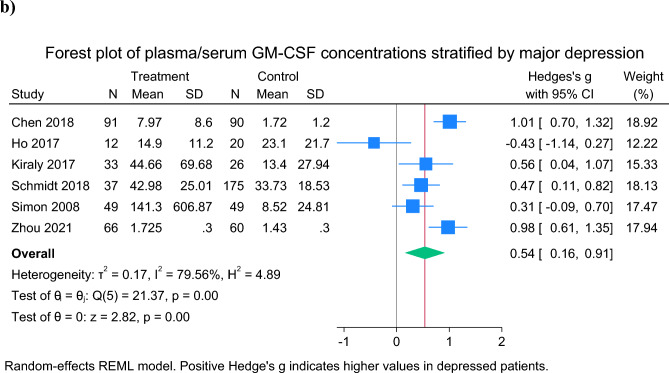
Forest plots of Th-17 measures in depressed patients versus healthy controls: **a** Interleukin-17 concentrations and **b** Granulocyte macrophage colony stimulating factor concentrations

Sixteen studies measured IL-17A [20–22, 29–42]. Compared to non-depressed controls, patients with depression showed increased plasma concentrations of IL-17A (SMD = 0.80 [95% CI 0.03 to 1.58], p = 0.042, N_studies_ = 11, Fig. 2a), though heterogeneity was very high (*I*^*2*^ = 96.7%). However, in random-effects meta-regression, antidepressant status was a significant moderator of the association between IL-17A and depression (Z = 2.12, p = 0.034). Even after exclusion of the obviously outlying study [34], the overall findings remained significant (SMD = 0.45 [95% CI 0.06 to 0.83], p = 0.023, N_studies_ = 10, *I*^*2*^ = 53.3%). There was evidence of small study effects (Egger’s test: Z = 3.02, p = 0.0025) (Supplementary Fig. 1a), though not after exclusion of the obvious outlying study (Egger’s test: Z = 0.83, p = 0.40). Of those studies not included in the meta-analysis due to between-group differences in age, sex or BMI, 3 studies reported higher IL-17A in depressed patient compared to controls [33, 36, 37], one reported higher IL-17A in controls [39], and one reported no difference [30]. A further study reported no difference between depressed and control groups [29], but this was not included in the meta-analysis due to lack of mean and SD data (Table 2).

**Table 2:** Summary of immune measures in depressed patients versus healthy controls

First author	Year	Between-group differences	GM-CSF	IL-5	IL-9	IL-13	IL-17	IL-23	IFN-γ	Th1 count	Th2 count	Th17 count
Al Hakeim	2020	Smoking							↑↑↑ Depressed			
Almulla	2023	BMI	↑ Depressed									
Alves	2020	Age, sex, smoking					↑↑↑ Depressed		↔			
Baek & Park	2013	None							↔			
Bayes	2023	BMI							↑ Controls			
Becking	2018	None										↑↑↑ Controls
Blzniewska-Kowalska	2020	Age					↑↑↑ Depressed					
Cassano	2017	BMI, smoking	↔	↑ Controls		↔			↔			
Chen	2018	None	↑↑↑ Depressed						↑↑↑ Depressed			
Chen	2011	None					↑ Depressed					↑↑↑ Depressed
Choi	2021	None							↔			
Daria	2020	None							↑↑↑ Controls			
Davami	2016	None					↑↑ Depressed					
Eidan	2019	Sex, smoking							↑↑↑ Depressed			
Elomaa	2012	None		↔		↔			↔			
Fanelli	2019	Age, sex, smoking	↔	↑ Controls			↑ Controls		↔			
Fornaro	2013	None							↑↑↑ Controls			
Ghosh	2020	None								↑ Controls		↑↑↑ Depressed
Grosse	2016	BMI, smoking								↔	↔	↑↑ Controls
Grosse	2016a	None										↔
He	2020	None	Not detected	↑ Depressed	↔	↔	↑↑↑ Depressed		↔			
Hernandez	2008	None				↑↑ Depressed			↑↑ Controls			
Ho	2017	None	↔	↔	↔	↑↑ Depressed	↔		↑ Controls			
Hocaoglu	2012	Age, sex							↔			
Hosseini	2017	None							↔			
Hughes	2012	None							↔			
Kageyama	2018	None	↔									
Kakeda	2018	Sex							↔			
Kim	2021	None					↑↑ Depressed					
Kim	2013	None					↔	↔				
Kim A	2007	None							↑↑ Controls			
Kiraly	2017	None	↑ Depressed				↔					
Lin	2018	None							↔			
Mao	2018	None				↑ Depressed						
Mao	2022	None					↑↑↑ Depressed					
Marques-Deak	2007	None							↔			
Mednova	2023	Sex				↑ Depressed	↔		↔			
Pavon	2006	None				↑ Depressed			↑ Controls			
Pedraz-Petronni	2020	None							↔			
Saraykar	2018	None					↔					
Schiweck	2020	BMI, smoking								↔	↔	↔
Schmidt	2018	None	Depressed	↑↑↑ Depressed		↑ Depressed			↑↑↑ Depressed			
Shelton	2015	Age					↔		↔			
Simon	2008	None	↑↑↑ Depressed	↔		↑↑ Depressed			↑↑↑ Depressed			
Spanemberg	2014	None					↔		↔			
Strawbridge	2019	None					↔		↔			
Suzuki	2017	BMI								↔	↔	↔
Syed	2018	Age, sex		↑↑↑ Depressed		↑↑↑ Depressed	↑↑↑ Depressed		↑↑↑ Depressed			
Wong	2018	None				↑ Controls						
Young	2016	None							↔			
Zincir	2016	None							↑↑↑ Depressed			
Zhou	2021	None	↑↑↑ Depressed	↔		↑ Depressed	↑↑ Depressed	↔	↔			
Zoga	2014	None							↑↑↑ Depressed			

Key: ↑ depressed = higher in depressed; ↑ controls = higher in controls, ↑ denotes p < 0.05 for difference, ↑↑ denotes p < 0.01 for difference, ↑↑↑ denotes p < 0.001 for difference, ↔ denotes no difference between groups

*GM-CSF* granulocyte macrophage colony stimulating factor, *IL* interleukin

### GM-CSF

GM-CSF was measured by 9 studies [21, 22, 39–41, 43–46]. Of these, 2 were not included in the meta-analysis due to differences between groups in age, sex or BMI; neither of these studies reported differences in GM-CSF between depressed patients and controls (Table 2) [39, 43]. In a further study, GM-CSF was not detectable in plasma [40]. In meta-analysis of the remaining 6 studies, patients with depression had higher GM-CSF concentations than controls (SMD = 0.54 [95% CI 0.16 to 0.91], p = 0.0047), although heterogeneity was high (*I*^*2*^ = 79.6%) (Fig. 2b).

### Th17 cell counts

Even studies measured Th17 cell counts [20, 47–52]. Of studies not included in meta-analysis due to between-group differences in age, sex or BMI, one study reported higher Th17 counts in controls [50], whilst another found no difference [51] (Table 2). In meta-analysis of the remaining 5 studies, patients with depression showed a non-significant trend towards higher Th17 cell counts (SMD = 0.44 [− 0.01 to 0.88], p = 0.06) across all studies, although heterogeneity was very high (*I*^*2*^ = 86.3%). All samples were unmedicated and one study adjusted its findings for age, sex and BMI [52]. No studies were low in quality.

### Other Th17 measures

Two studies measured IL-23 and found no difference between depressed patients and controls [22, 29]. Two studies measured CXCL-1: one reported lower concentrations in depressed patients [39], but this was an elderly sample in which depressed patients were older and more likely to be female than controls. Another study reported higher CXCL1 concentrations in depressed patients, though there were between-group differences in BMI [53] (Table 2). No study measured IL-22.

## Th1 *axis*

### Th1 cell counts

In meta-analysis of 4 studies [48, 49, 51, 52], there was no difference between Th1 cell counts between patents with depression and controls (SMD = − 0.19 [95% CI − 0.47 to 0.085], p = 0.17, *I*^*2*^ = 61.7%). One of these studies had pre-adjusted its findings for age, sex and BMI [52].

### IFN-gamma

**Fig. 3:**
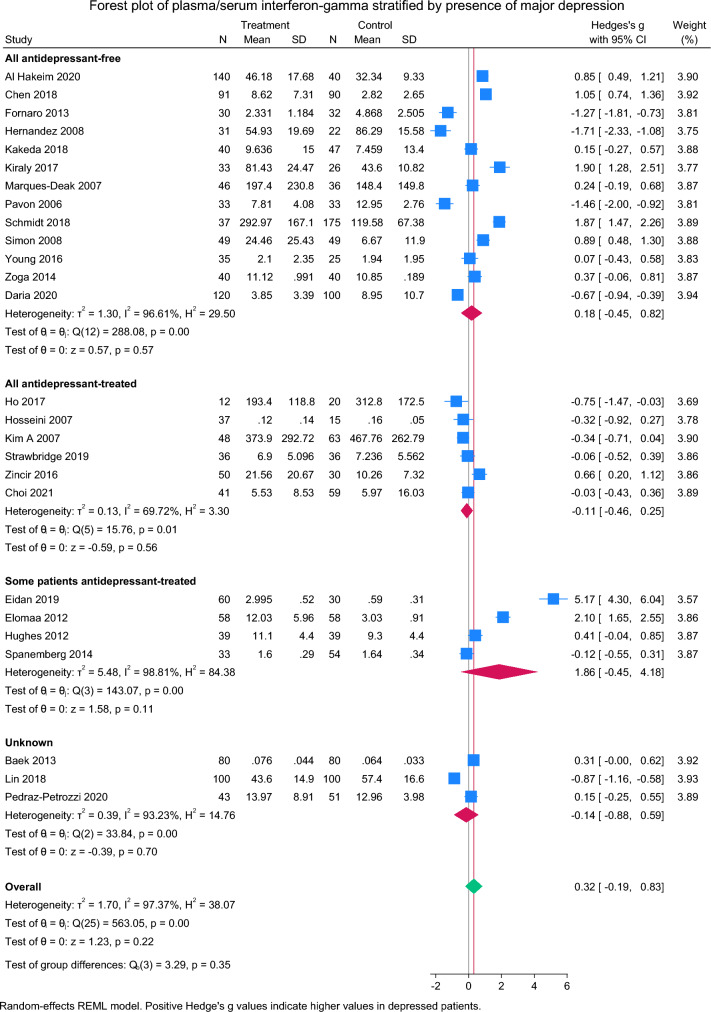
Forest plot of interferon-gamma concentrations in depressed patients versus healthy controls

Thirty-four studies compared IFN-γ between depressed patients and controls [30–33, 36, 39–41, 43–46, 53–73]. Of 8 studies not included in meta-analysis due to between-group differences in age, sex or BMI, 2 reported higher IFN-γ concentrations in depression [33, 59], 1 found higher concentrations in controls [73], and 8 found no differences between depressed patients and controls [30, 36, 39, 43, 53, 62, 65] (Table 2). In meta-analysis of the remaining 26 studies, there was no overall difference in IFN-γ concentration between depressed and non-depressed patients (SMD = 0.32 [95% CI − 0.19 to 0.83, p = 0.22]), although heterogeneity was very high (*I*^*2*^ = 97.4%) (Fig. 3). Egger’s test of small study effects was negative (Z = 1.51, p = 0.13) (Supplementary Fig. 1b). In analysing only unmedicated samples, there remained no significant difference in IFN-γ by depression status (SMD = 0.18 [95% CI − 0.45 to 0.82], p = 0.57, *I*^*2*^ = 96.6%, N_studies_ = 13). Prescription of any antidepressants did not moderate the association between depression and IFN-γ concentrations (Z = − 0.18, p = 0.86). In sensitivity analysis, there remained no significant difference after exclusion of small studies (SMD = 0.36 [95% CI − 0.16 to 0.88], p = 0.18, *I*^*2*^ = 97.5%, N_studies_ = 25) or poor-quality studies (SMD = 0.46 [95% CI − 0.09 to 1.01], *I*^*2*^ = 97.4%, p = 0.10, N_studies_ = 23).

## Th2 *axis*

### Th2 cell counts

In meta-analysis of 3 studies [49, 51, 52], Th2 cell counts were similar in patients with depression compared to controls (SMD = 0.003 [95% CI − 0.17 to 0.18], p = 0.97, *I*^2^ = 0%). No studies were of low quality and all patients were untreated with antidepressants.

### IL-13

**Fig. 4:**
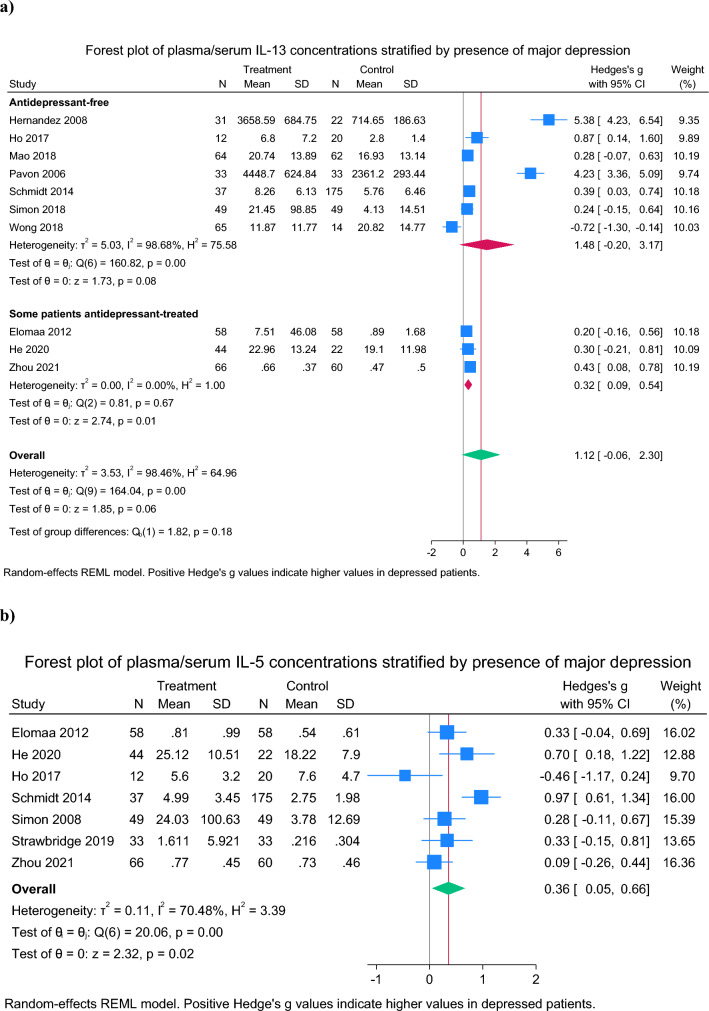
Forest plots of Th2 measures in depressed patients versus healthy controls: **a** Interleukin-13 concentrations and **b** Interleukin-5 concentrations

Thirteen studies measured IL-13 [22, 33, 40, 41, 43, 46, 53, 58, 61, 74–76]. Of those not included in the meta-analysis, one reported higher IL-13 in the depressed group [33] while two reported no difference [43, 53] (Table 2). In meta-analysis of the remaining 10 studies [40, 41, 46, 58, 61, 74–76], there was no difference between patients and controls in IL-13 concentrations (SMD = 1.12 [95% CI − 0.06 to 2.30], p = 0.063), although there was extremely high between-study heterogeneity (98.5%) (Fig. 4a) and there was evidence of small study effects (Z = 4.68, p < 0.001) (Supplementary Fig. 1c). After excluding one low-quality study, the findings remained non-significant (SMD = 1.32 [95% CI − 0.16 to 2.80]). In a subgroup analysis by antidepressant treatment, the findings were unchanged in who were antidepressant-free. Indeed, prescription of any antidepressant did not moderate the findings (Z = − 0.87, p = 0.38). Only one study [40] was fully matched a priori for age, sex and BMI.

### IL-5

Eleven studies measured IL-5 [22, 31, 33, 39–41, 43, 45, 46, 53, 58]. Of those not included in the meta-analysis due to between-group differences in age, sex or BMI, one reported higher IL-5 in depressed patients [33], two found higher IL-5 in controls [39, 43], and one found no difference [53] (Table 2). In meta-analysis of the remaining 7 studies, depressed patients had higher IL-5 compared to controls (SMD = 0.36 [95% CI 0.05 to 0.66] , p = 0.02, *I*^2^ = 70.5%) (Fig. 4b). After exclusion of small studies, the findings remained significant (SMD = 0.52 [95% CI 0.24 to 0.81], *I*^*2*^ = 56.6%). No studies were low in quality. In unmedicated patients, the findings were no longer significant (SMD = 0.31 [95% CI − 0.48 to 1.09], p = 0.44, *I*^2^ = 88.4%, N_studies_ = 3), likely reflecting a loss of statistical power.

## Th9 *axis*

### IL-9

Two studies measured IL-9 but neither reported significant differences between depressed patients and controls [40, 41] (Table 2). A third study also reported no difference, though there were between-group differences in BMI [53].

## Discussion

In this systematic review and meta-analysis, we examined whether activation of the IL-23/Th17 axis—using plasma/serum measures—is associated with major depression. We adopted a pan-pathway approach, measuring the cytokine (IL-23) that activates the Th17 cell, the Th17 count itself, and the key cytokines and chemokines secreted by Th17 cells, all in relation to depression. To assess specificity of the IL-23/Th17 axis, we also measured T-helper cell counts and key cytokines on the Th1, Th2 and Th9 axes between patients with depression and healthy controls. We found higher plasma concentrations of key cytokines secreted by Th17 cells (IL-17A and GM-CSF) in patients with depression compared to healthy controls. We also found preliminary evidence that antidepressant treatment may modify the association between IL-17A and depression. There was also an association between Th2 activation and depression, as estimated using plasma IL-5, although findings for IL-13, another Th2 cytokine, were inconsistent. By contrast, we found no association between Th1 and Th9 cell activation and depression, as estimated using the T-helper cell counts and canonical cytokines secreted by these cells.

### Comparison with previous literature

Whilst there have been previous meta-analyses of inflammatory markers in depression, these have typically captured a wide range of cytokines but with limited understanding of the upstream immune pathways driving these.

To our knowledge, our key findings are original. For example, our finding of higher IL-17A concentrations in depression contrast the negative results of a 2017 meta-analysis, which was based on only 3 studies [77]. Furthermore, a more recent meta-analysis did not look at IL-17A [27] and neither meta-analysis extracted data for GM-CSF. There has been no previous meta-analysis of Th17 cell counts in depression, as authors have generally focussed on cytokines or chemokines only. Our positive finding for IL-5 is also novel and contrasts the negative findings of the 2020 meta-analysis, which was based on 4 studies rather than the 6 in our synthesis. Our negative findings for IL-13 and IFN-γ are consistent with previous studies [27].

A striking, albeit preliminary, finding was that antidepressants moderated the association between IL-17A and depression, which is also unreported in any previous meta-analysis. Although this finding should be cautioned by the low number of studies involved, these results have face validity, as SSRI antidepressants have been found to reduce plasma concentrations of IL-17A [34, 78].

Our findings complement a growing mechanistic and genetic literature suggesting activation of the IL-23/Th17 axis in depression and neuroinflammation. For example, single nucleotide polymorphisms (SNP) on the IL-23 axis are associated with depressive symptoms in the general population [79, 80]. Th17 cells and IL-17A have been found to increase the permeability of the blood–brain-barrier and induce neuroinflammation [81, 82].

The aetiology of IL-23/Th17 activation in depression is not understood, though gut dysbiosis (changes in the gut microbiome) is a conceptually attractive potential upstream regulator, since the community composition of intestinal microbiota, including the presence or absence of particular mucosal-associated species, critically shapes Th17 responses [83]. Similarly, *Roseburia* species regulate Th17-dependent immune responses [84] and are reduced in abundance in patients with Crohn’s disease who have depression [85]. In pre-clinical models, Th17 cells have been found interact with the gut microbiome to promote depressive-like behaviours [86]. This suggests that the IL-23/Th17 axis could be a key substrate of the gut-brain-axis, providing a link between gut dysbiosis and depression.

### Clinical implications

To date, broad-based anti-inflammatory approaches have produced inconsistent benefits for major depression [6–9]. More targeted, higher-potency anti-inflammatory approaches, such as anti-TNF therapy, have shown limited promise [7, 10, 11] and are associated with potentially serious side-effects even within their licensed populations [12]. A major advantage of the IL-23/Th17 axis is the wide range and safety of the medications that can be used to modify it. Within conditions such as IBD and psoriasis, treatments that modify the IL-23 could be used preferentially in patients who have comorbid depression, which is highly prevalent in these populations. Our findings also support pilot/feasibility studies testing the repositioning of anti-IL-23/17 treatments for major depression in the general population, for example in patients with treatment-resistant depression. Furthermore, there is a need for both parenteral and long-acting treatments for major depression, for example in in patients who are poorly compliant with antidepressants or in patients with poor gut absorption. Anti-IL-23 treatments can be administered every 6–8 weeks, providing a potential long-acting therapy for major depression that does not require gut absorption. Finally, probiotic therapies may help to regulate the IL-23/Th17 axis [87] and are a promising novel therapy for major depression [88]. Future research should test whether probiotics may improve major depression by regulating Th-17-dependent pathways.

### Strengths and limitations

Our findings are strengthened by the hypothesis-driven, pan-pathway approach to the IL-23/Th17 axis, which reduced multiple testing biases. We also assessed specificity for the IL-17 axis by comparing it against T-helper cell counts and canonical cytokines for other key immune pathways. We only included studies that used diagnostic criteria for major depression. Recognizing the potential for confounding effects of age and sex on immune measures, we only included studies in meta-analysis that lacked between-group differences in these covariates, and we further excluded from meta-analysis any studies with known between-group differences in BMI. Our findings are limited by significant heterogeneity in some of the findings, particularly for interferon-gamma. The cross-sectional analysis means that causality cannot be established. There was evidence of small-study effects for the IL-17A findings, highlighting the need for larger observational studies. Data for some key measures, such as IL-22, were insufficient for meta-analysis.

### Summary

In this systematic review and meta-analysis, we found that key measures on the IL-23/Th17 axis—including plasma/serum IL-17A and GM-CSF—were elevated in patients with major depression compared to non-depressed controls. We further found evidence that antidepressant treatment moderated the association between IL-17A and depression. Further research is needed to explore the IL-23/Th17 axis in depression, including up-stream mechanisms that may drive the association, as well as intervention studies testing the IL-23/Th17 as a modifiable target for depression in inflammatory diseases and in the general population.

## Supplementary Information

Below is the link to the electronic supplementary material.

Supplementary file1 (DOCX 71 KB)

## Data Availability

The data from this paper will be made available by the corresponding author upon reasonable request.
