# Supplementary material for: Activation of the interleukin-23/Th17 axis in major depression: a systematic review and meta-analysis
Source: Eur Arch Psychiatry Clin Neurosci. 2024 Jul 16;275(6):1653–73. doi: 10.1007/s00406-024-01864-2 (PMC12500783; doi:10.1007/s00406-024-01864-2)
Supplement: Supplementary file 1 — Supplementary file1 (DOCX 71 KB) [file 406_2024_1864_MOESM1_ESM.docx]

# SUPPLEMENTARY APPENDIX 1: Newcastle Ottawa Quality assessment scale for case control studies

**SELECTION**

**1) Is the case definition adequate?**

*a) yes, with independent validation (1 point)*

b) yes, e.g. record linkage or based on self-reports

c) no description

**2) Representativeness of the cases**

*a) consecutive or obviously representative series of cases (1 point)*

b) potential for selection biases or not stated

**3) Selection of Controls**

*a) community controls (1 point)*

b) hospital controls

c) no description

**4) Definition of Controls**

*a) no history of psychiatric or medical disease (1 point)*

b) any history of psychiatric or medical comorbidity

**COMPARABILITY**

**1) Comparability of cases and controls based on the design or analysis**

*a) study controls for age (1 point)*

*b) study controls for BMI (1 point)*

**EXPOSURE: immune factor measurement**

**1) Assay**

*a) specifies assay type, brand/model (1 point)*

*b) standardised protocol for sample collection (1 point)*

e) no description or no standardised protocol

**2) Same method of ascertainment for cases and controls**

*a) yes (1 point)*

b) no

| **Supplementary Table 1: Modified Newcastle-Ottawa quality scoring of included studies** | | | | | | | | | | | |
| --- | --- | --- | --- | --- | --- | --- | --- | --- | --- | --- | --- |
| Study author |  | **Selection** | | | | **Comparability** |  | **Exposure** | | |  |
|  | Year | Adequate case definition | Cases representative | Selection of controls | Definition of controls | Matched *a priori* for age | Matched *a priori* for BMI | Standardised assay for measuring inflammatory markers | Following a standardised process for sample collection | **Total score (/8)** | Rating (poor, fair, good) |
| Al Hakeim | 2020 | 1 | 1 | 0 | 1 | 1 | 1 | 1 | 1 | 7 | Good |
| Almulla | 2023 | 1 | 0 | 1 | 1 | 0 | 0 | 1 | 1 | 4 | Fair |
| Alves | 2020 | 1 | 0 | 0 | 1 | 1 | 1 | 1 | 1 | 6 | Fair |
| Baek & Park | 2013 | 1 | 0 | 0 | 1 | 1 | 1 | 1 | 1 | 6 | Fair |
| Bayes | 2024 | 1 | 0 | 0 | 1 | 1 | 0 | 1 | 1 | 5 | Fair |
| Becking | 2018 | 1 | 0 | 0 | 1 | 1 | 0 | 1 | 0 | 4 | Fair |
| Blzniewska-Kowalska | 2020 | 1 | 1 | 1 | 0 | 0 | 1 | 1 | 0 | 5 | Good |
| Cassano | 2017 | 1 | 1 | 1 | 1 | 1 | 0 | 1 | 1 | 7 | Good |
| Chen | 2018 | 1 | 0 | 1 | 1 | 1 | 1 | 1 | 1 | 7 | Good |
| Chen | 2011 | 1 | 0 | 0 | 1 | 1 | 0 | 1 | 1 | 5 | Fair |
| Choi | 2021 | 1 | 0 | 0 | 1 | 1 | 1 | 1 | 1 | 6 | Fair |
| Daria | 2020 | 1 | 1 | 0 | 1 | 1 | 1 | 1 | 0 | 6 | Good |
| Davami | 2016 | 1 | 0 | 0 | 1 | 1 | 1 | 1 | 0 | 5 | Fair |
| Eidan | 2019 | 1 | 0 | 0 | 0 | 1 | 1 | 1 | 1 | 5 | Fair |
| Elomaa | 2012 | 1 | 1 | 1 | 1 | 1 | 0 | 1 | 0 | 6 | Good |
| Fanelli | 2019 | 1 | 1 | 1 | 0 | 1 | 1 | 1 | 0 | 6 | Good |
| Fornaro | 2013 | 1 | 0 | 0 | 1 | 0 | 0 | 1 | 1 | 4 | Poor |
| Ghosh | 2020 | 1 | 0 | 0 | 1 | 1 | 0 | 1 | 0 | 4 | Fair |
| Grosse | 2016 | 1 | 0 | 1 | 1 | 1 | 1 | 1 | 0 | 6 | Good |
| Grosse | 2016a | 1 | 0 | 1 | 1 | 1 | 0 | 1 | 1 | 6 | Good |
| He | 2020 | 1 | 0 | 0 | 1 | 0 | 0 | 1 | 0 | 3 | Poor |
| Hernandez | 2008 | 1 | 0 | 1 | 1 | 1 | 1 | 1 | 1 | 7 | Good |
| Ho | 2017 | 1 | 0 | 1 | 1 | 1 | 1 | 1 | 1 | 7 | Good |
| Hocaoglu | 2012 | 1 | 0 | 0 | 1 | 1 | 1 | 1 | 1 | 6 | Fair |
| Hosseini | 2007 | 1 | 0 | 1 | 0 | 1 | 0 | 1 | 1 | 5 | Fair |
| Hughes | 2012 | 1 | 0 | 1 | 1 | 1 | 1 | 1 | 0 | 6 | Good |
| Kageyama | 2018 | 1 | 0 | 1 | 1 | 1 | 0 | 1 | 0 | 5 | Good |
| Kakeda | 2018 | 1 | 0 | 1 | 1 | 1 | 0 | 1 | 0 | 5 | Good |
| Kim | 2021 | 1 | 0 | 1 | 1 | 1 | 0 | 1 | 0 | 5 | Good |
| Kim | 2013 | 1 | 0 | 1 | 1 | 1 | 1 | 1 | 1 | 7 | Good |
| Kim A | 2007 | 1 | 0 | 0 | 1 | 1 | 1 | 1 | 1 | 5 | Fair |
| Kiraly | 2017 | 1 | 0 | 0 | 1 | 1 | 1 | 1 | 1 | 6 | Fair |
| Lin | 2018 | 1 | 0 | 0 | 1 | 0 | 0 | 1 | 0 | 3 | Poor |
| Mao | 2018 | 1 | 0 | 1 | 1 | 1 | 1 | 1 | 1 | 7 | Good |
| Mao | 2022 | 1 | 0 | 0 | 1 | 1 | 0 | 1 | 1 | 5 | Fair |
| Marques-Deak | 2007 | 1 | 0 | 0 | 1 | 1 | 0 | 1 | 1 | 5 | Fair |
| Pavon | 2006 | 1 | 0 | 1 | 1 | 1 | 0 | 1 | 1 | 6 | Good |
| Pedraz-Petronni | 2020 | 1 | 0 | 1 | 1 | 1 | 1 | 1 | 1 | 7 | Good |
| Saraykar | 2018 | 1 | 0 | 1 | 1 | 0 | 0 | 1 | 0 | 4 | Poor |
| Schiweck | 2020 | 1 | 0 | 0 | 1 | 1 | 1 | 1 | 0 | 5 | Fair |
| Schmidt | 2018 | 1 | 0 | 0 | 1 | 1 | 1 | 1 | 0 | 5 | Fair |
| Shelton | 2015 | 1 | 0 | 0 | 1 | 1 | 1 | 1 | 0 | 5 | Fair |
| Simon | 2008 | 1 | 1 | 1 | 1 | 1 | 0 | 1 | 0 | 6 | Good |
| Spanemberg | 2014 | 1 | 0 | 0 | 1 | 0 | 0 | 1 | 0 | 3 | Poor |
| Strawbridge | 2019 | 1 | 0 | 1 | 0 | 1 | 1 | 1 | 0 | 5 | Fair |
| Suzuki | 2017 | 1 | 0 | 1 | 1 | 1 | 1 | 1 | 0 | 6 | Good |
| Syed | 2018 | 1 | 0 | 0 | 1 | 1 | 1 | 1 | 0 | 5 | Fair |
| Wong | 2008 | 1 | 0 | 1 | 1 | 1 | 1 | 1 | 1 | 7 | Good |
| Young | 2016 | 1 | 0 | 0 | 1 | 1 | 1 | 1 | 1 | 6 | Fair |
| Zincir | 2016 | 1 | 0 | 0 | 1 | 1 | 0 | 1 | 1 | 5 | Fair |
| Zhou | 2021 | 1 | 0 | 0 | 1 | 1 | 0 | 1 | 1 | 6 | Fair |
| Zoga | 2014 | 1 | 0 | 0 | 1 | 1 | 1 | 1 | 1 | 6 | Fair |

**Supplementary Figure 1**

A)

B)

C)
